# C-955-02. Burn Surgery with a Lighter Footprint: The First Case-Level Carbon Emission Study in Burns

**DOI:** 10.1093/jbcr/irag033.151

**Published:** 2026-04-06

**Authors:** Mohammad R Goodarzi, Alexandra L F Sutcliffe, Hajar S Abdulla, Dharminder Dhillon, Christopher J Lewis

**Affiliations:** Northern Regional Burn Centre, Newcastle Upon Tyne, England; Northern Regional Burn Centre, Newcastle Upon Tyne, England; Royal Victoria Infirmary, Newcastle, England; Royal Victoria Infirmary, Newcastle, England; Northern Regional Burn Centre, Newcastle Upon Tyne, England

## Abstract

**Introduction:**

Operating theatres represent a major carbon hotspot in healthcare. Despite burn surgery being uniquely resource-intensive, its carbon footprint has never been measured at case level. This study provides the first case-level quantification of emissions in burns surgery, identifies its main drivers, and highlights opportunities for emission reduction.

**Methods:**

A prospective observational study was conducted at a regional burns centre. For each case, total body surface area (TBSA), procedure type, consumables, anaesthesia, and energy proxies were recorded. Carbon footprint (kg CO₂e) was calculated using published emission factors. Linear regression evaluated the association between TBSA and emissions. Mann–Whitney U tests compared grafting with non-grafting, and volatile anaesthesia with total intravenous anaesthesia (TIVA). Multivariable linear regression identified independent predictors of emissions.

**Results:**

Twenty-eight consecutive adult procedures were analysed. Burn size ranged from 0.25 to 85% TBSA (mean 7.9%). Mean footprint was 251 kg CO₂e per case (range: 49-922). Emissions exceeded laparoscopic surgery (20-60 kg), were similar to caesarean section or joint replacement (100-300 kg), and were lower than cardiac surgery (450-800 kg). Consumables accounted for 69% of emissions, followed by anaesthesia (18%) and theatre energy (13%). Grafting produced threefold higher emissions than non-grafting cases (*p*<.01). Emissions increased linearly with burn size (R^2^ = 0.75, *p*<.001), rising by around 9 kg per 1% TBSA. Longer operative time and use of complex single-use kits (e.g., fibrin sealant spray or spray-on, autologous skin cell suspension) independently increased emissions (*p*<.01).

**Conclusions:**

Burn surgery has a substantial carbon footprint that scales with burn size, grafting, and consumable intensity. On average, one burn operation equated to the footprint of driving ~800 miles in a petrol car. The largest share of emissions arose from single-use consumables — gowns, drapes, gloves, dressings, and high-impact kits such as fibrin sealant spray and spray-on, autologous skin cell suspension. Unlike theatre energy or case complexity, these are largely modifiable and emissions can be reduced by rationalising their use. Other improvements include adopting reusable gowns and drapes, streamlining theatre time, and improving energy efficiency. Anaesthesia contributed less in this cohort due to exclusive use of sevoflurane and TIVA, but strict avoidance of high carbon footprint inhaled agents such as desflurane and nitrous oxide remains critical.

**Applicability of Research to Practice:**

This first case-level analysis highlights practical targets for greener burn care. By tackling consumables, theatre energy, and anaesthetic choice, burns services can cut emissions while maintaining safety. These findings provide the first quantitative benchmark for burns surgery and inform international net-zero agendas.

**Funding for the Study:**

N/A.

